# Building and breaking the gut barrier with bariatric surgery

**DOI:** 10.15698/cst2022.02.263

**Published:** 2021-12-22

**Authors:** Mohammed K. Hankir

**Affiliations:** 1 Department of Experimental Surgery, University Hospital Wuerzburg, Wuerzburg, 97080, Germany.

**Keywords:** bariatric surgery, gut barrier, gut microbiota, insulin resistance, endotoxemia

## Abstract

Bariatric surgery has been proposed to improve glycemic control in morbidly obese patients by stabilising the gut barrier and alleviating endotoxemia-induced insulin resistance. Here, recent studies are highlighted which reveal site-specific and at times opposing effects of bariatric surgery on the gut barrier. Further understanding the underlying mechanisms may not only inform the development of novel gut-based drugs for the initial treatment of type 2 diabetes, but possibly also assist in the management of its eventual relapse.

The gut lumen harbors trillions of microbiota which normally reside symbiotically in the host. Under certain conditions however, such as in inflammatory bowel disease (IBD), this healthy balance is lost, and there is an expansion of pro-pathogenic gut microbiota which disrupt the otherwise protective gut barrier. The resulting translocation of microbial products such as lipopolysaccaride (LPS) from the cell wall of gram-negative bacteria through ’leaky’ gut epithelial cells into the general circulation is referred to as endotoxemia. This is characterized by both a localised and systemic low-grade inflammatory response via the activation of toll-like receptor 4 (TLR4) expressed in innate and adaptive immune cells. In 2008, it was proposed based on experiments on mice that obesity-associated insulin resistance can be traced to microbiota-mediated disruption of the gut barrier and the ensuing endotoxemia [1]. This idea has since been substantiated in many other preclinical studies and, more importantly, has been translated to humans [2]. Thus, gut barrier-stabilising interventions such as gut microbiota transplant [3] or small molecule drugs [4] are being considered as novel anti-diabetic treatments.

Bariatric surgeries such as vertical sleeve gastrectomy (VSG) and Roux-en-Y gastric bypass (RYGB) are indicated for individuals with morbid obesity when conservative treatments fail. These gastrointestinal anatomy-modifying interventions not only cause unmatched weight loss, but also a host of other metabolic benefits such as the remission of type 2 diabetes in most cases. Endotoxemia is also alleviated in patients after VSG and RYGB [5], although it remains to be shown if this is necessary for the postoperative improvements in glycemic control. Nevertheless, several studies in robust rodent models are beginning to reveal complex, site-specific effects of bariatric surgery on the gut barrier. Here, I discuss these studies in detail and propose that understanding the mechanisms behind both the positive and the negative impact bariatric surgery has on the gut barrier could inform the design of novel gut-based drugs for the initial treatment and potentially also the eventual relapse of type 2 diabetes.

In the first study by Blanchard *et al.* [6], mildly diet-induced obese mice that underwent VSG experienced weight loss and had reduced food intake as well as improved oral glucose tolerance compared with sham-operated controls - all important features of the procedure in patients. Having established the validity of their VSG model, the authors next addressed the potential site-specific effects of VSG on the gut barrier by performing *ex vivo* functional and gene expression analyses of jejunal and colonic tissue samples. They found that jejunal samples from the VSG-treated mice had reduced paracellular and transcellular permeability as determined by sulfonic acid and horse radish peroxidase (HRP) fluxes, respectively. Interestingly, this was not found in jejunal samples from mice pair-fed to VSG-treated mice arguing for a surgery-specific effect. In line with the functional findings, jejunal mRNA expression of several gut barrier-associated proteins including junctional adhesion molecule-A and occludin were selectively upregulated in VSG-treated mice. The authors then strikingly found the opposite pattern in the colon. That is, VSG-treated mice had increased transcellular and paracellular colonic permeability although this was not associated with any changes in colonic gene expression. Perhaps unexpectedly, but in line with the greater bacterial load present in the large compared with the small intestine, systemic endotoxemia was increased specifically in VSG-treated mice. While these findings are at odds with the outcome of VSG on endotoxemia in patients described above [5], they provide an early example of how manipulating gastrointestinal anatomy can have opposing, site-specific effects on the gut barrier. They are also in line with findings in patients after VSG showing improvements in gastroduodenal permeability as determined by urinary recovery of ingested sucrose and worsening of colonic permeability as determined by urinary recovery of ingested sucralose [7].

In the second study by Jin *et al*. [8], severely diet-induced obese mice received RYGB and experienced weight loss as well as markedly improved oral glucose tolerance and insulin sensitivity compared with sham-operated controls. The authors next addressed the potential site-specific effects of RYGB on the gut barrier by performing protein expression analyses of jejunal and colonic tissue samples in conjunction with gold-standard *in vivo* functional experiments. It was found that protein expression of occludin was clearly increased in the jejunum of RYGB-treated mice, while protein expression of the gut barrier-associated protein zona occludin-1 was clearly increased in the colon. Nevertheless, jejunal and colonic permeability were similarly decreased in RYGB-treated mice as determined by localised fluorescein isothiocyanatedextran injections. Correspondingly, endotoxemia was alleviated in these mice as it is in humans after RYGB thus providing confidence that this feature of the clinical procedure can effectively be modeled in the preclinical setting. Next, the authors investigated the potential site-specific effects of RYGB on gut inflammation. They found that mRNA expression of the proinflammatory cytokine interleukin 1 beta (IL-1β) decreased in the jejunum of RYGB-treated mice, while mRNA expression of pro-inflammatory IL-6 decreased in the colon. This was also reflected in the circulating levels of both cytokines, confirming that RYGB mitigates the state of chronic low-grade systemic inflammation associated with obesity. In order to identify the underlying, site-specific mechanisms for the alleviation of endotoxemia associated with RYGB, a different approach was taken from previous studies and gene expression analysis was performed on factors involved in host defense in jejunal and colonic samples. It was found that mRNA expression of the Paneth cell-derived antimicrobial factor regenerating islet-derived protein 3 beta (REG3β) increased in the jejunum, while mRNA expression of both REG3β and REG3γ increased in the colon of RYGB-treated mice. In contrast, mRNA expression of the enzyme intestinal alkaline phosphatase (which dephosphorylates LPS) increased in both the jejunum and colon of these mice. While these insightful findings are only associational in nature, they uniquely suggest that RYGB creates a gut environment that is hostile to pathogenic bacteria and effectively neutralizes LPS alongside reducing gut permeability. They are also in line with the findings of Blanchard et al [6] of site-specific effects of bariatric surgery on the gut barrier, although in this instance they are both favourable.

Given the above discoveries, one might ask what accounts for the site-specific effects of bariatric surgery on the gut barrier? This is unlikely to be a circulating factor acting on the basolateral side of enterocytes. Instead, it is more likely that luminal factors acting on the apical side of enterocytes are responsible considering how reconfiguration of gastrointestinal anatomy imposes site-specific compositional changes in the gut lumen due to redirection of digestive secretions and shifts in the gut microbiota. To directly test this idea, Hankir *et al*. [9] applied the duodenal, jejunal and colonic content of RYGB-treated and sham-operated rats onto Caco-2 cells, a human intestinal epithelial cell line, to in effect simulate the intestinal microenvironments of the respective groups. Remarkably, it was found that content from all of these gut regions of RYGB-treated rats had marked barrier-stabilising effects, and that duodenal and colonic content required the farnesoid X receptor, a nuclear receptor for bile acids, to do so. Next, to test if gut microbiota-derived factors are required for the barrier-stabilising effects of gut content from RYGB-treated rats, transfer experiments were performed on germ-free mice. It was found that jejunal and colonic, but not duodenal, content of these rats improved glycemic control in association with attenuated endotoxemia. Collectively, these results suggest that primary bile acids mediate the gut barrier-stabilising effects of duodenal content and secondary bile acids mediate the but barrier-stabilising effects of colonic content of RYGB-treated rats (**Figure 1**). In contrast, because of the diversion of bile flow away from the jejunum, as yet unidentified host- and/or microbiota-derived metabolites mediate the gut barrier-stabilising effects of jejunal content of RYGB-treated rats (**Figure 1**).

**Figure 1:**
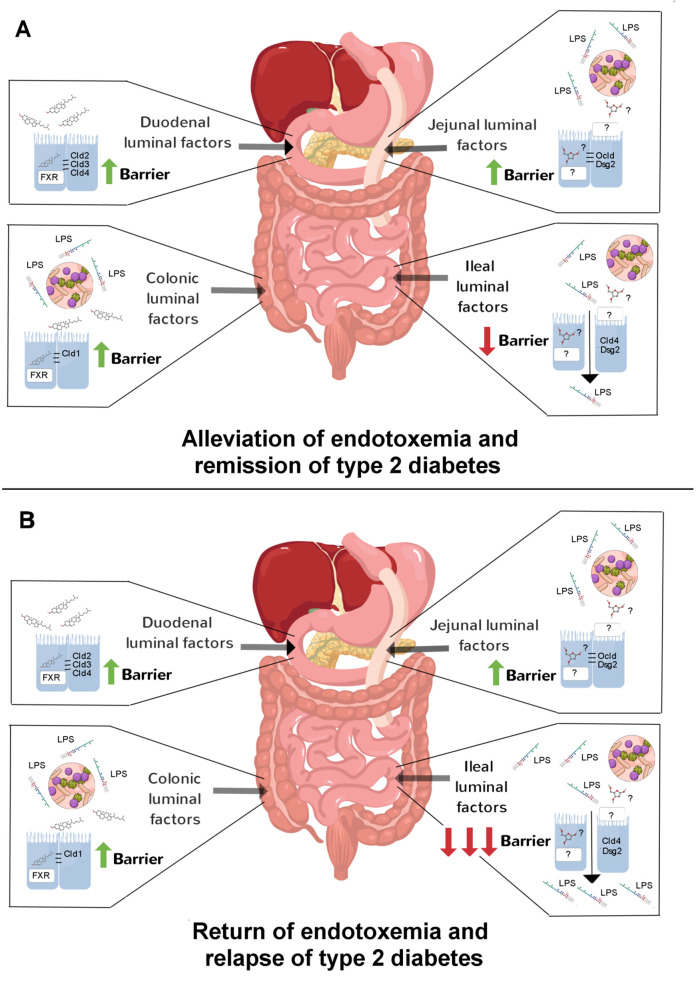
FIGURE 1: Postulated contribution of site-specific effects of RYGB on the gut barrier on glycemic control. **(A)** During diabetes remission after RYGB, the barrier-stabilising effects of jejunal and colonic content may outweigh the barrier-destabilising effects of ileal content leading to alleviation of endotoxemia-induced insulin resistance. **(B)** During diabetes relapse, the barrier-destabilising effects of ileal content may outweigh the barrier-stabilising effects of jejunal and colonic content leading to a return of endotoxemia-induced insulin resistance.

In a subsequent study by Hankir *et al*. [10], content from the remaining section of the gut, i.e. the ileum, of RYGB-treated and sham-operated rats was investigated. Reminiscent of the studies of Blanchard *et al*. [6] and Jin *et al*. [8], ileal content of RYGB-treated rats caused the opposite effect of content from the other gut regions. That is, it increased paracellular permeability in Caco-2 cells and heightend endotoxemia in germ-free mice [10]. The former effect was associated with a strong reduction in the tight junction protein claudin-4 and the adherens junction protein desmoglein-2 [10] in Caco-2 cells. The implications of these findings are unclear, but raise the possiblity that during relapse of type 2 diabetes in the significant proportion of patients after RYGB the barrier-disruptive ileal microbiota expand thereby exerting a dominant (negative) effect on endotoxemia (**Figure 1**). This would be analogous to the situation in mice after VSG where enhanced colonic permeability dictated endotoxemia development [6]. It would be interesting in future studies to identify the microbiota and their metabolites in the ileal content of RYGB-treated rats that destabilise the gut barrier and to test if their neutralization leads to even greater and lengthier benefits on glycemic control. Such an approach may even assist in the management of the relapse of type 2 diabetes in patients after RYGB although at present this remains only speculative.

Initially considered as simply mechanical interventions, bariatric surgeries are now recognized as having complex molecular, cellular and systems effects. This is reflected nicely in the context of a leaky gut, where physically cutting and then merging the gastrointestinal tract leads to widespread and site-specific changes in the molecular building blocks of the gut barrier that overall alleviate endotoxemia-induced insuin resistance. Future studies can reveal whether designing drugs that target the gut barrier in the same way as bariatric sugeries do can effectively treat type 2 diabetes in a safe and non-invasive manner.

## References

[1] Cani PD, Bibiloni R, Knauf C, Waget A, Neyrinck AM, Delzenne NM, Burcelin R (2008). Changes in gut microbiota control metabolic endotoxemia-induced inflammation in high-fat diet-induced obesity and diabetes in mice.. Diabetes.

[2] Chakaroun RM, Massier L, Kovacs P (2020). Gut Microbiome, Intestinal Permeability, and Tissue Bacteria in Metabolic Disease: Perpetrators or Bystanders?. Nutrients.

[3] Depommier C, Everard A, Druart C, Plovier H, Van Hul M, Vieira-Silva S, Falony G, Raes J, Maiter D, Delzenne NM, de Barsy M, Loumaye A, Hermans MP, Thissen JP, de Vos WM, Cani PD (2019). Supplementation with Akkermansia muciniphila in overweight and obese human volunteers: a proof-of-concept exploratory study.. Nat Med.

[4] Natividad JM, Agus A, Planchais J, Lamas B, Jarry AC, Martin R, Michel ML, Chong-Nguyen C, Roussel R, Straube M, Jegou S, McQuitty C, Le Gall M, da Costa G, Lecornet E, Michaudel C, Modoux M, Glodt J, Bridonneau C, Sovran B, Dupraz L, Bado A, Richard ML, Langella P, Hansel B, Launay JM, Xavier RJ, Duboc H, Sokol H (2018). Impaired Aryl Hydrocarbon Receptor Ligand Production by the Gut Microbiota Is a Key Factor in Metabolic Syndrome.. Cell Metab.

[5] Yang PJ, Lee WJ, Tseng PH, Lee PH, Lin MT, Yang WS (2014). Bariatric surgery decreased the serum level of an endotoxin-associated marker: lipopolysaccharide-binding protein.. Surg Obes Relat Dis.

[6] Blanchard C, Moreau F, Chevalier J, Ayer A, Garcon D, Arnaud L, Pais de Barros JP, Gautier T, Neunlist M, Cariou B, Le May C (2017). Sleeve Gastrectomy Alters Intestinal Permeability in Diet-Induced Obese Mice.. Obes Surg.

[7] Kellerer T, Brandl B, Büttner J, Lagkouvardos I, Hauner H, Skurk T (2019). Impact of Laparoscopic Sleeve Gastrectomy on Gut Permeability in Morbidly Obese Subjects.. Obes Surg.

[8] Jin Z, Chen K, Zhou Z, Peng W, Liu W (2021). Roux-en-Y gastric bypass potentially improved intestinal permeability by regulating gut innate immunity in diet-induced obese mice.. Sci Rep.

[9] Hankir MK, Langseder T, Bankoglu EE, Ghoreishi Y, Dischinger U, Kurlbaum M, Kroiss M, Otto C, le Roux CW, Arora T, Seyfried F, Schlegel N (2020). Simulating the Post-gastric Bypass Intestinal Microenvironment Uncovers a Barrier-Stabilizing Role for FXR.. iScience.

[10] Hankir MK, Seyfried F, Schellinger IN, Schlegel N, Arora T (2021). Leaky Gut as a Potential Culprit for the Paradoxical Dysglycemic Response to Gastric Bypass-Associated Ileal Microbiota.. Metabolites.

